# The Experimental Verification of Direct-Write Silver Conductive Grid and ARIMA Time Series Analysis for Crack Propagation

**DOI:** 10.3390/s21206916

**Published:** 2021-10-19

**Authors:** Artur Kurnyta, Marta Baran, Paulina Kurnyta-Mazurek, Kamil Kowalczyk, Michał Dziendzikowski, Krzysztof Dragan

**Affiliations:** 1 Airworthiness Division, Air Force Institute of Technology, 01-494 Warsaw, Poland; marta.baran@itwl.pl (M.B.); kamil.kowalczyk@itwl.pl (K.K.); michal.dziendzikowski@itwl.pl (M.D.); krzysztof.dragan@itwl.pl (K.D.); 2 Faculty of Mechatronics, Armament and Aerospace, Military University of Technology, 00-908 Warsaw, Poland; paulina.mazurek@wat.edu.pl

**Keywords:** crack detection, SHM, conductive paint, silver microparticles, structural integrity, fatigue test, time series analysis, ARIMA modeling

## Abstract

The paper presents experimental verification of customized resistive crack propagation sensors as an alternative method for other common structural health monitoring (SHM) techniques. Most of these are sensitive to changes in the sensor network configuration and a baseline dataset must be collected for the analysis of the structure condition. Sensors investigated within the paper are manufactured by the direct-write process with electrically conductive, silver-microparticle-filled paint to prepare a tailored measuring grid on an epoxy or polyurethane coating as a driving/insulating layer. This method is designed to enhance the functionality and usability compared to commercially available crack gauges. By using paint with conductive metal particles, the shape of the sensor measuring grid can be more easily adapted to the structure, while, in the previous approach, only a few grid-fixed sensors are available. A fatigue test on the compact tension (CT) specimen is presented and discussed to evaluate the ability of the developed sensors to detect and monitor fatigue cracks. Additionally, the ARIMA time series algorithm is developed both for monitoring and predicting crack growth, based on the acquired data. The proposed sensors’ verification reveal their good performance to detect and monitor fatigue fractures with a relatively low measurement error and ARIMA estimated crack length compared with the crack opening displacement (COD) gauge.

## 1. Introduction

At present, aircrafts are, in general, maintained by periodic inspection with the use of traditional nondestructive testing techniques (NDT) such as eddy current, ultrasound, radiography, and thermograph methods, etc. There is often the need for at least the partial disassembly of the structure during these inspections, which is a difficult and time-consuming task. Additionally, it is impossible to access some particular areas of the aircraft airframe, in which cracks are likely to generate or form during long-term operational use. For this reason, monitoring solutions permanently integrated with the structure are being researched and developed to overcome these limitations [1].

Structural health monitoring (SHM) is an umbrella term that refers to cutting-edge technologies aimed at developing systems capable of the continuous, not periodic, monitoring of structural elements for fatigue damage. The concept implies the use of permanently attached sensor networks and minimal human intervention to increase safety as well as reduce the life-cycle costs of the technical object. The SHM measuring methods are partly based on a number of different well-established NDT techniques, such as ultrasonic inspection or eddy current methods. The evolution of the SHM systems began with desirable intended functions, e.g., crack detection, corrosion detection, operational load monitoring, etc. Nowadays, development efforts are driven by general requirements or demands from commercial or military platforms, such as:

- Demands for reductions in maintenance costs (reductions in inspection times, reductions in inspection efforts, improvements in inspection quality, etc.);
- Demands for certifiable SHM systems that enable underwriting new aircraft materials and designs;
- Demands for improvements in operational management, affordability, and performance.

In order to address the above, the potential intended functions of an SHM system should provide improved knowledge in detecting various types of damage as well as maintenance, operational, and/or ownership benefits, such as reducing inspection costs and times, improving repair planning, optimizing inspection intervals, reducing aircraft downtime, increasing residual values of used aircraft, reducing weight, and enabling life extensions [2,3].

### 1.1. The State-of-the-Art

Currently, several candidate sensor technologies suitable for SHM application have been widely investigated through lab-scale experiments. They include active and passive piezoelectric transducers, e.g., lead zirconate titanate (also called PZT) ceramics [4,5,6], fiber-optic and Fiber Bragg Gratings (FBG) [7,8], strain gauges [9,10], comparative vacuum monitoring (CVM) [11,12], and foil eddy current sensors [13,14]. Some of the SHM technologies have matured in recent years, allowing them to be tested during full-scale fatigue tests and experimental flights, or have already been installed in civil airplanes in service (FBG, CVM, PZT).

At the same time, new techniques that differ from NDT-derived methods have been developed to monitor structures for cracks or flaws. Most of them are based on new capacitive and resistive sensors developed with materials such as allotropes of carbon (e.g., nanotubes, graphite, graphene) or metal micro/nanoparticles (e.g., silver, copper) in the form of pastes or inks ready for printing on the driving layer by various manufacturing methods. In [15,16], a water–ethanol base formulation with nanosized copper or pulsed bias arc ion Cu plating on a dielectric layer was applied on a specimen under a fatigue test. The change in the electric field of the conductive sensing layer was achieved as a fluctuation in the measured electrical potential value, due to crack initiation and propagation on the sensor coating induced by a structural crack. A grid-pattern ion-sputtered gold film was proposed in [17] to measure the crack length of a specimen during a bending test. The shape of the sensing grid was very dense; as a result, even very short, micro-sized cracks were detected with high precision. An empirical analytical equation was proposed to move from voltage sensor information to crack length. In [18], the application of an electrically conductive silver thin film, with the use of electrical impedance spectroscopy to detect the time, location, and approximate number of cracks on the surface of cementitious materials, was described. The arrangement of multiple side electrodes enabled the detection of several cracks within the applied film sensor. Furthermore, in [19] and [20], carbon nanotubes were investigated to develop electrically conductive patterns to detect fatigue cracks. The so-called “smart sensing skin” for structural health monitoring applications was investigated [20]. The sensor was a thin-film soft elastomeric capacitor (SEC) that transduced strain into a measurable change in capacitance. Arranged in a network configuration, the SEC had the capability of detecting and localizing damage by detecting local deformation over a global surface. A styrene–ethylene–butylene–styrene (SEBS) copolymer and carbon black particle compound was utilized for sensor fabrication. The performance of the SEC at detecting and localizing fatigue cracks in steel structures was investigated and confirmed.

Other technologies under investigation, especially for composite structures, are gaining attention, including the use of the self-sensing capabilities of carbon-fiber-reinforced polymers (CPRF) or nanofiller-modified composites (also known as nanocomposites) [21,22]. The application of a glass-fiber-reinforced polymer (GFRP), with a nanoparticle-modified matrix by a dispersion of different filler contents of, e.g., multi-walled carbon nanotubes and carbon black, was investigated in [23]. Using in-plane and through-thickness electrical resistance measurements, the impact detection of GFRP as well as its surface deformation and damage were monitored. In this case, the matrix was therefore used as a sensor material. Electrical resistance measurements allowed for the detection of inter- and intralaminar matrix failure and the indirect detection of fiber failure in FRP [24] with a high potential resolution.

### 1.2. Customized Resistive Crack Propagation Sensors

**Figure 1:**
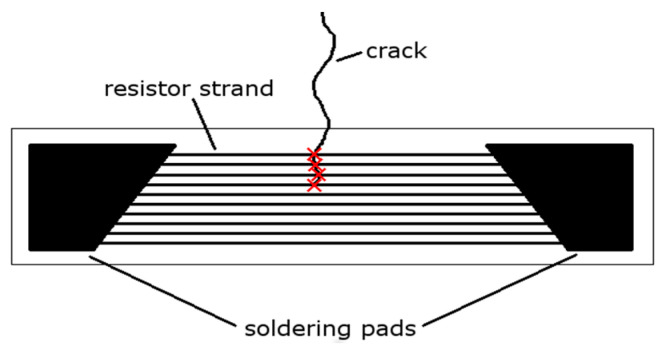
An example of a commercially available crack gauge.

The customized resistive crack propagation sensors proposed in this article could become a good alternative method for crack detection and monitoring, due to their economic and functional features, which will be discussed further. Fatigue crack detection and its size determination are the basic aims of the SHM system. One of the approaches to fatigue crack monitoring is the use of commercially available crack gauges, as shown in Figure 1.

The sensor is composed of a series of conductive strands connected in parallel. When a crack propagates underneath the sensor bonded to the structure, subsequent strands of the sensor break down. This results in a stepwise increase in the sensor’s resistance. Therefore, by tracking the resistance of the sensor, the condition of the structure can be inferred. Investigation of this technique was performed for both coupons, realistic structures [25], and during a full-scale fatigue test [26] of an aircraft structure. The method can provide a convenient, straightforward, and economically effective solution to indicate the presence of a crack and to quantify its linear size in a predetermined location on a test part or structure. The method of integrating the sensor into the structure is similar to that for regular foil strain gauges, so the surface needs to be mechanically and chemically prepared before bonding to ensure good and reliable results.

However, due to the material used for the sensing grid of the commercially available crack gauges (mainly constantan), they are usually unidirectional, and the course of the crack propagation must be known a priori. Additionally, there are only a few sensor shapes in commercial off-the-shelf (COTS) products. For this reason, they are difficult to use in areas with the dense occurrence of, e.g., rivets, on narrow wing spars or frames, etc.

**Figure 2:**
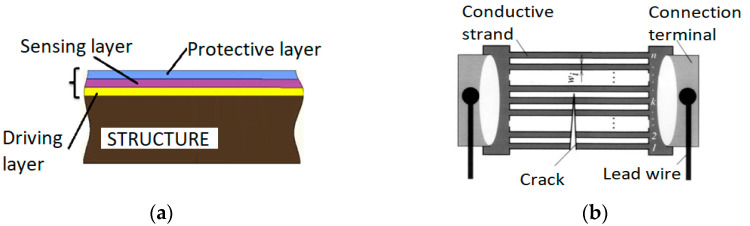
The crack propagation sensor: (**a**) cross-section; (**b**) top view.

The solution proposed in this paper is a customized resistive crack propagation sensor, which can overcome the limitations and drawbacks of typical crack gauges and allow the tailoring of the shape of the sensor’s measuring grid to the specific structure. The feature of adapting the measuring grid shape translates directly into the ability to detect even relatively short cracks (sensor installed closer to the “hot spot”) and damage size quantification. Additionally, the overall dimensions of the sensor itself can be adjusted to fit the specific location or structural element. Customized resistive crack propagation sensors are fabricated using flexible insulating materials for the driving and protective layers and conductive paint with silver microparticles for the sensing layer (Figure 2). Compared to most methods mentioned in the previous subchapter, the sensor’s conductive layer is not continuous, which allows for the discrete but precise evaluation of the crack length, even in the presence of external factors (e.g., temperature, load).

A manufactured gauge is able to follow the propagating crack after being connected to a low-level DC power source and voltage signal acquisition unit. However, even a straight digital multimeter (DMM) is suitable for integration for the periodic verification of the sensor’s resistance change. The use of the conductive paint to fabricate the sensing grid, in contrast to commercially available crack gauges, enables at least two means of sensor integration with the structure. First, the sensor can be executed in situ layer-by-layer directly on the structure, or it can be pre-fabricated and bonded in a second approach. In both cases, the measuring grid shape can be quickly customized to the monitored structure to increase its crack detection capabilities. Several mechanical and electrical differences can be noticed between these two sensor manufacturing and integration methods. Sensitivity to crack occurrence as well as differences between true and measured damage size due to the driving layer thickness are essential.

Sensor pre-fabrication is a convenient method for the automated printing of a measuring grid with precise and complex shapes on a driving layer. The manufacturing process also ensures high repeatability and uniformity of conductive strands within the sensing layer, so the output resistance characteristic of the sensor can be estimated with a close tolerance. Unfortunately, such devices are not suitable for portable utilization, and the sensor performance can suffer when poorly customized to the “hot spot” location. The driving layer thickness is increased, and an additional bonding layer is present–both could decrease the measurement sensitivity for fatigue crack development.

**Figure 3:**
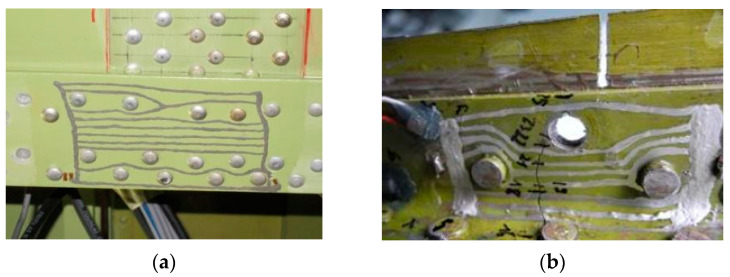
The customized crack propagation sensors, fabricated directly on the structure: (**a**) the bottom central wing section during full-scale fatigue test, (**b**) a fragment of a wing spar during lab test.

Direct-write (e.g., by soft lithography, drawing pen, spatula, etc.) manufacturing methods are characterized by better permanent integration with the structure, lower thickness of the sensor layers, and a lack of additional bonded joints. Another benefit is that the sensor can be fully customized to the “hot spot” location, and the strands of the sensing layer can be placed as closely as possible to the potential crack propagation area (Figure 3). However, forming a measuring grid in poorly accessible areas is a challenging task for staff. In addition, precise, complex shapes of the measuring grid with both uniformity and repeatability for individual strand execution may be difficult to obtain. Both affect the achievement of the intended output characteristics of the sensor.

## 2. Materials and Methods

### 2.1. Crack Propagation Sensors as Silver Conductive Grid

**Figure 4:**
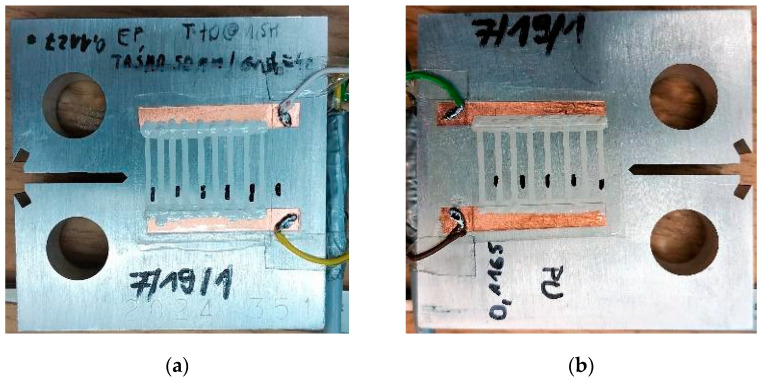
A view of two sides of CT coupon specimen with customized crack propagation sensors: (**a**) epoxy (EP), (**b**) polyurethane (PU) driving layer.

The experimental verification of direct-write customized resistive crack propagation sensors for the detection and quantification of fatigue cracks was investigated by performing a laboratory fatigue test. During the test, a compact tension specimen (CT coupon) was used, with 2 simply shaped sensors installed on both sides of the specimen, as shown in Figure 4. The simple shape of the sensing layer was chosen for this test as a proof-of-concept and a direct analogy to commercially available crack gauges, although a more complex grid can be achieved, as shown in Figure 3.

Sensors were fabricated layer-by-layer on the specimen, using translucent two-part epoxy (832C, MG Chemicals, Ontario, Canada) on one side and 1-part polyurethane coating (M-Coat A, Vishay Precision Group, Malvern, PA, USA) on the other side of the CT coupon as a driving and top layer (Figure 4). Tape masking and a doctor blade were used to prepare and smooth the driving layers, which were cured for 1.5 h at 70 degrees Celsius in the oven. As a sensing grid layer material, Pelco^®^ 16031 Colloidal Silver Paint (Ted Pella, Inc., Redding, CA, USA) was applied on the driving layer with a rapidograph drawing pen and left to cure at ambient temperature for more than 24 h. Side terminals for wire connections were produced using thin copper adhesive tape.

For the measurement of the shape of the conductive grids and to check the subjective quality of the sensor layers, an optical microscope, the Keyence VHX-6000 (Keyence International, Belgium), was utilized. The distance between the CT coupon notch and the individual stand, single strand width, separation of adjacent strands, and overall sensing layer area were measured. The lack of heterogeneity in the driving and sensing layers, single strand edge area smoothness, and its continuity were evaluated before the fatigue test.

**Figure 5:**
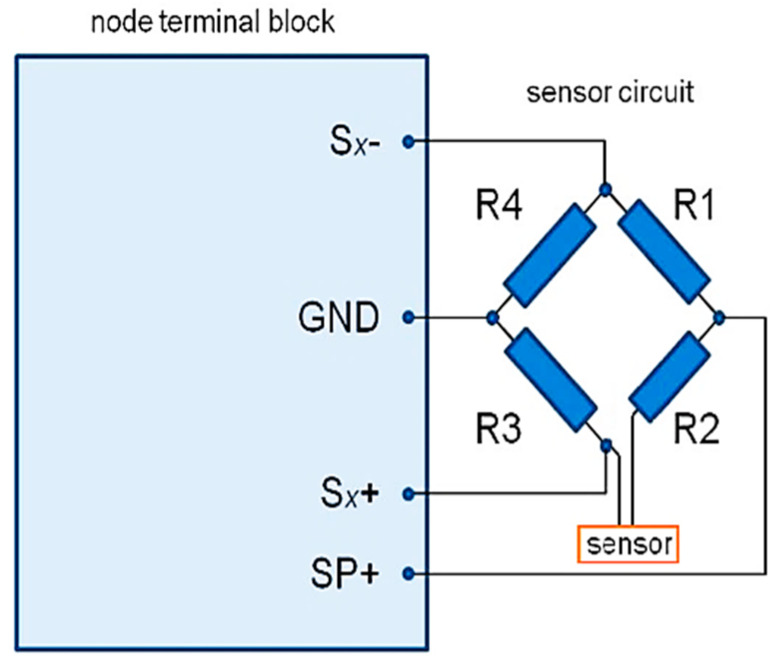
The measurement circuit for crack propagation sensors.

During the execution of the fatigue test, the sensors were connected to the differential voltage input of the wireless V-Link-200 sensor node (Lord MicroStrain^®^ Sensing, Williston, VT, USA) through a Wheatstone bridge as a basic electrical circuit. The bridge was completed with 350 ohm resistors, one in each arm, and the additional resistance (crack propagation sensor) was placed in one arm (Figure 5). Raw data from the sensors were pre-processed for further analysis using the DIAdem data management software (NI, Austin, TX, USA), and this included resampling from 64 Hz to 60 Hz (for synchronization with data from the fatigue machine) and smooth filtering (3 point width median filter).

### 2.2. Test Procedure and Additional Instrumentation of Fatigue Machine

**Figure 6:**
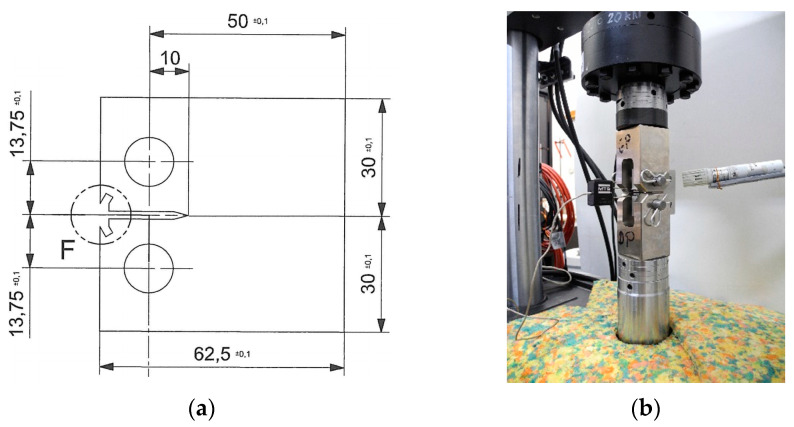
The CT coupon specimen for fatigue test: (**a**) dimensions; (**b**) installed in test machine with COD mounted at front.

The specimen was cut from a 13 mm thick sheet of EN AW-2024 T351 alloy (*Rm* = 461 MPa, *Re*_0,2_ = 317 MPa) and its geometry is shown in Figure 6a.

The fatigue crack growth rate test was conducted according to the ASTM E647-15ε1 standard using a servo-hydraulic testing system (Landmark test system, MTS, Eden Prairie, MN, USA) and software provided by the manufacturer, i.e., 790.40 Fatigue Crack Growth. The crack size was determined based on a compliance method using a crack opening displacement (COD) gauge (632.03F-30 model, MTS, Eden Prairie, MN, USA). The following parameters were kept constant during the entire test: stress ratio *R* = 0.1, frequency *f* = 30 Hz, and sinusoidal waveform of control signal. Crack length measurement during the test was additionally realized using a compliance method, in which a correlation between the compliance of the specimen and the length of the crack was applied. Both the compliance and the crack length in the correlation equation were dimensionless, i.e., the compliance (*c*) is expressed by a reciprocal of the force (*P*)–displacement (*v*) slope normalized for an elastic modulus (*E*) and a specimen thickness (*B*)

(1) E*v*B/P=c,

Displacement (*v*) refers to displacement between the measurement points in front of the specimen, marked in Figure 6a with dashed circles. The specimen fitted in the fatigue machine and mounted on the COD gauge for displacement measurement is depicted in Figure 6b.

Meanwhile, the dimensionless crack length was obtained by crack length *a* divided by the specimen width (*W*). In the case of the compact specimen, the crack length *a* was defined by the distance from a reference plane containing bearing points of force application to the specimen to the crack front (which means that a machined notch length *a_n_* is included in the magnitude of the crack length *a*).

The equation of the correlation of the tested specimen had the following form:

(2) α=aw=C0+C1*ux+C2*ux2+C3*ux3+C4*ux4+C5*ux5,

where:

(3) $$u_{x}={\left\{{\left[\frac{E*v*B}{P}\right]}^{\frac{1}{2}}+1\right\}}^{-1},$$

and the coefficients of equation *C_i_* were set empirically.

### 2.3. Crack Prediction with ARIMA Modeling

For the test presented above, the autoregressive integrated moving average (ARIMA) model to predict crack propagation was used, based on data registered during the experiment from the crack propagation sensors. The dedicated model was selected for the obtained time series.

The ARIMA model, also called the Box–Jenkins model, is widely used in monitoring and control systems to estimate future values of univariate time series [27]. It consists of three components: the autoregressive process AR, moving average MA, and degree of integration I.

The autoregressive component uses the process memory and estimates the current value as a linear weighted sum of previous values of time series [27]. The AR element is characterized by the order marked as *p*. The order determines how many previous values have an effect on the current value of the process. The AR component is described by the following equation:

(4) $$y_{t}=\rho_{1}y_{t-1}+\rho_{2}y_{t-2}+\ldots +\rho_{p}y_{t-p}+\varepsilon_{t},\;\mathrm{for}\;t=1,2,\ldots ,p,$$

where *y_t_* is the time series value at time *t* (predicted value) and *y_t_*_−1_, *y_t_*_−2_, …, *y_t−p_* are time series values at times *t* − 1, *t* − 2, …, *t* − *p*. In turn, *ε_t_* is a random component, indicating disorder at time *t*. The coefficients *ρ*_1_, *ρ*_2_, …, *ρ_p_* are parameters of the AR process and show the influence of previous series values on the current time series value [28].

Further, the moving average process MA is similar to the autoregressive one. In this case, the current values of the time series depend on the disorder at time *t*, and at times *t* − 1, *t* − 2, …, *t* − *q*. Parameter *q* is the order of the MA process, which is expressed by the following equation:

(5) $$y_{t}=\beta_{1}\varepsilon_{t-1}+\beta_{2}\varepsilon_{t-2}+\ldots +\beta_{q}\varepsilon_{t-q}+\varepsilon_{t},\;\mathrm{for}\;t=1,2,\ldots ,q$$

where coefficients *β*_1_, *β*_2_, …, *β_q_* are parameters of the MA process and show the influence of previous disorder values on the current time series value.

The last component of the ARIMA model is integration I. It is characterized by parameter *d*, which shows the integration degree of the time series. This ARIMA component enables the time series process to be reduced to a stationary form.

In summary, the ARIMA model has three parameters, which must be defined during the modeling process: order of AR element—*p*, order of MA component—*q*, and integration degree—*d*.

The prediction of crack propagation was realized with the use of Matlab software. The *arima*(*p*,*d*,*q*) function was used to create a univariate ARIMA model of voltage time series, [29,30]. During the modeling process, three input arguments (parameters) *p*, *d*, and *q* were specified, which took values of nonnegative integers and were described previously.

For the purpose of the following research, which was the verification of the effectiveness and applicability of ARIMA modeling in the prediction process of crack propagation, we assumed a simple model with *p* = 1, *d* = 1, and *q* =1. The designed algorithm also uses the *estimate* and *forecast* Matlab functions. Determination of the ARIMA model and the prediction of future values of the time series was carried out iteratively. In each iteration, 1000 future series values were predicted based on almost 20,000 samples.

## 3. Results

### 3.1. Continuous Crack Propagation Monitoring Test

The fatigue test was performed with the use of the MTS 370.10 fatigue machine. It was divided into two parts with force excitation of 30 Hz: (i) with a decreasing stress intensity factor for approx. 2.5 million cycles; (ii) with a constant amplitude force until the specimen was broken due to the fatigue load. Before the main part of the test, the calibration procedure of COD and the pre-cracking for the CT specimen were conducted, which are beyond the scope of the paper.

**Figure 7:**
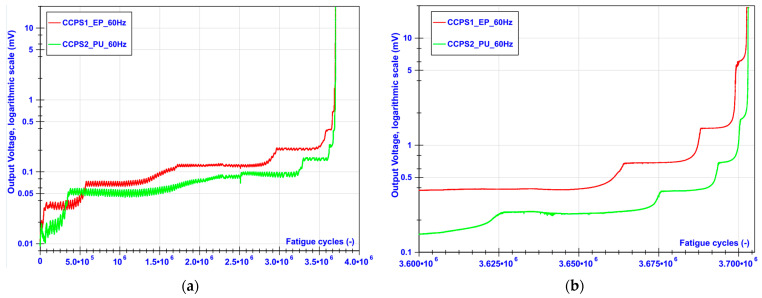
The output voltage from customized crack propagation sensors during CT coupon fatigue test: (**a**) the whole test, (**b**) last 100,000 cycles with high crack growth rate.

The measured output voltage from sensors on both sides of the CT coupon are illustrated in Figure 7. The CCPS1_EP conductive grid was fabricated on the epoxy resin, and CCPS2_PU on the polyurethane coating. Due to high value of the crack growth rate at the end of the test, the data from last 100,000 cycles are magnified in Figure 7b to reveal the sensors’ performance in this region.

Similarly to classical crack gauges, the stepwise output characteristic could be seen as the crack was propagated during the test. Signal steps at the beginning of the crack evolution were small but stable. Signal fluctuations, mainly seen at the initial stage of the test, were caused by temperature drifts around the test stand. Then, the signal steps became evident, when the number of broken strands was increased. Both driving layer materials—epoxy (EP) as well as polyurethane (PU)—showed good insulation between the conductive grids and the specimen and also the adhesion to its structure during the fatigue test. Slightly higher signal fluctuations could be observed for the PU layer. This can be explained by the incompatibility of the thermal expansion coefficient of the PU driving layer and the specimen or the elasto-plastic deformation of this type of driving layer. The PU was found to be more flexible than EP, and its hardness was within the Shore A range, while epoxy was considered Shore D. Nevertheless, for both sensors, the crack was transferred from the specimen to the sensor layers equally. The monotonic increase in the signals between stable step values confirms that the single conductive strand tore apart gradually with the coupon.

**Figure 8:**
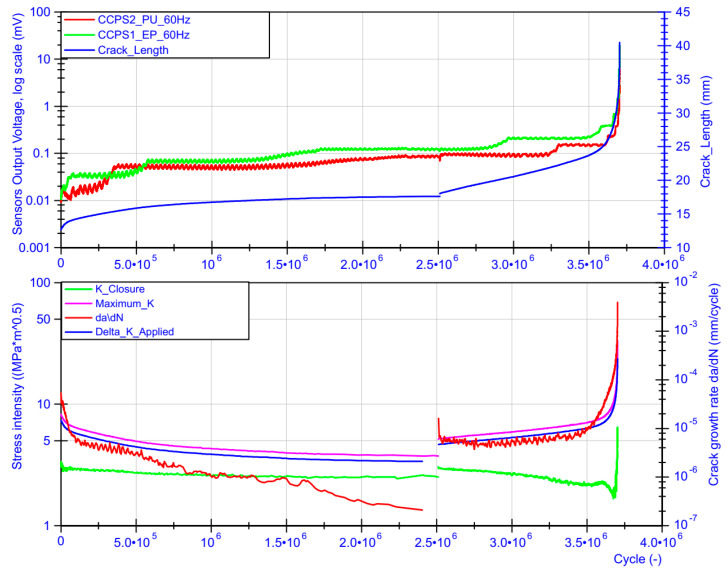
Performance of conductive grids due to change in fatigue test parameters.

Figure 8 collectively presents data from the conductive grids as well as from the fatigue machine controller. The crack length from the upper chart was measured by the COD gauge, with the reference at the fitting points of the CT specimen. As shown in Figure 6a, it was 10 mm. The starting value included approx. 2.6 mm of the pre-crack. During the first part of the test, the decreasing stress intensity factor Δ*K_Applied_* (named Delta_K_Applied in Figure 8) from 7.3 to 3.4 MPa $\sqrt{m}$ caused the same trend in the crack growth rate da/dN. For this reason, longer periods of maintaining the stable voltage level from the conductive grids were observed, as well as a wider range of the transient state while the strand of the conductive grid was torn apart. In the constant amplitude force part of the test, a linear increase in crack length as well as the stress intensity factor could be observed for the COD gauge measurements from 18 to 24 mm. The crack growth rate decreased its value from 1.61 × 10^−5^ mm/cycle and reached a valley at 4.40 × 10^−6^ around approx. 2,900,000 cycles. After this, both parameters da/dN and Δ*K_Applied_* increased linearly, reaching 1.61 × 10^−5^ mm/cycle around 3,500,000 cycles of the test. Further rapid acceleration of crack propagation could be noticed for all parameters in Figure 8 until the CT specimen became permanently damaged. Immediately before the end of the test, the crack growth rate as well as stress intensity factor reached their maximum values of 3.92 × 10^−3^ mm/cycle and 23.6 MPa $\sqrt{m}$, respectively. In the region of the high value of Δ*K_Applied_*, the effect of plasticity started to influence the crack growth rate because the plastic zone size became large before the crack tip. As can be observed in Figure 7b, even in this region, signals from the silver conductive grids showed a highly stable stepping characteristic between transient states due to the tearing of the silver strand.

**Figure 9:**
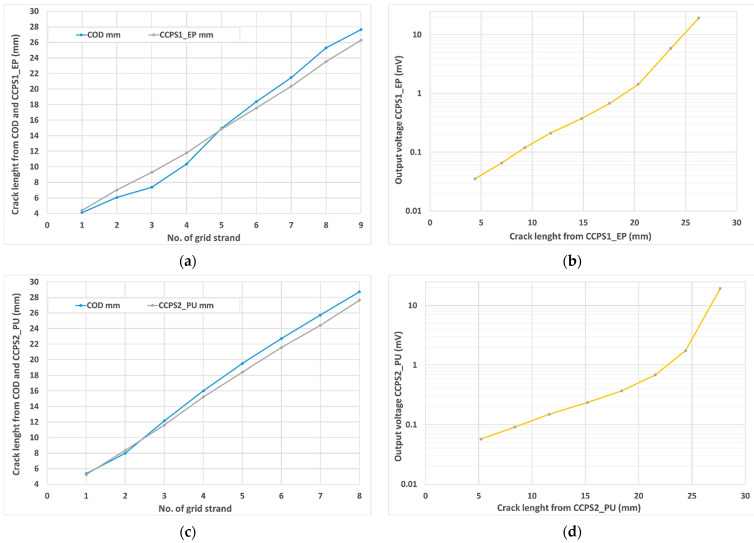
(**a**) The crack length measurement comparison from COD and sensor CCPS1_EP; (**b**) crack length versus output voltage for sensor CCPS1_EP; (**c**) crack length measurement comparison from COD and sensor CCPS2_PU; (**d**) crack length versus output voltage for sensor CCPS2_PU.

The comparison of crack lengths measured by the COD gauge and predefined conductive grid shapes (strand separation) is presented in Figure 9a for CCPS_EP and in Figure 9c. The shape of both sensors’ grids, the single strand distances from the specimen notch, and their widths were measured before conducting the test by the optical microscope. The corresponding crack length from the COD was captured when the sensor voltage signal stabilized (reached the next stable step value) after the transient state (Figure 8—top). Additionally, for both sensors, CCPS1_EP and CCPS2_PU, the characteristics of output voltage as a function of the crack length are shown in Figure 9b,d, respectively.

It can be observed that for sensor CCPS2_PU with the polyurethane driving layer, the crack length measured by the COD gauge and optical image were similar for the first three strands. After this, the crack length from the COD gauge was approx. 1–1.5 mm longer than measured by the conductive grid. The same can be seen for the CCPS1_EP sensor on the epoxy driving layer after the sixth strand was torn apart. The reason for the decreased crack length value for both sensors compared to COD will be considered in a further investigation. There could be several potential causes, e.g., the thickness of the driving layer, a delay in strain transfer from the specimen surface to the sensing layer, or local delamination of the sensor near the crack plastic zone, or, on the other hand, slight overestimation of the COD indication in the higher Δ*K_Applied_* region. Nonetheless, the offset between the COD and crack propagation sensors seems to be stable and acceptable in operational application.

**Figure 10:**
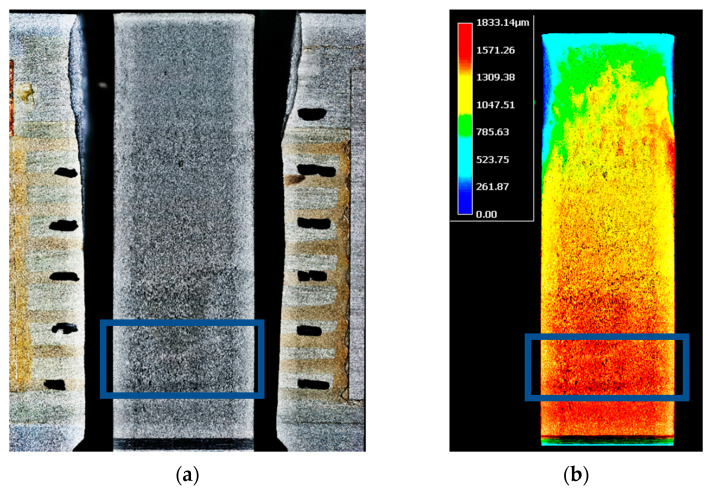
The microscope images of the specimen after test: (**a**) combined images of sensor CCPS2_PU (left), fracture surface (center), sensor CCPS1_EP (right); (**b**) fracture surface height profile from 3D optical scan mode.

However, some differences in the measured crack length were also seen for the CCPS1_EP sensor for the first four strands of the conductive grid. While, for the first strand, this offset was negligible (0.27 mm), for the second, third, and fourth strand, the conductive grid showed, respectively, 0.96 mm, 1.90 mm, and 1.42 mm higher crack lengths than the COD gauge. There could be at least two main causes that explain this phenomenon. The first is the false positive indication of the crack propagation from the sensor due to the low fatigue strength of the EP layer. As a consequence, the sensor could have suffered from adhesive failure and become delaminated from the host structure of the CT specimen. It is less likely that the force acting on the specimen was higher than the cohesion force of the EP layer, because, during this stage of the fatigue test, the stress intensity factor as well as the excitation force were constantly decreasing. The second explanation could be uneven crack propagation in relation to the CT coupon thickness (which was 13 mm). In other words, the front of the crack was not parallel to the line of the notch. If so, the surface cracks on the sidewalls of the specimen would not have had equal length. The microscope images of the specimen taken after test are shown in Figure 10 to verify the fracture surface through the thickness of the CT coupon. As revealed by Figure 10a, the darker shade of the fracture seems to start and finish lower on the left (side of the CCSP2_PU) than on the right (side of the CCSP1_EP). The difference is at least 1 mm. The same can be confirmed by the fracture surface height profile in Figure 10b, indicated by the dark red area at the bottom of the image (marked by blue frame). The uneven crack propagation through the specimen thickness could be caused by, e.g., the movement of the test article in the clamps, the machining of the notch while preparing the specimen, or fatigue machine piston misalignment. However, one of the main indicators of the correctness of the compliance test is the crack propagation in the center of the specimen. The height profile scan shows that the change in height along the direction of crack propagation in the fatigue fracture range was less than 0.8 mm. Thus, it can be concluded that the differences in the crack length measured by the COD gauge and the sensor CCPS1_EP grid were due to uneven propagation in the specimen thickness.

### 3.2. ARIMA Time Series Analysis

**Figure 11:**
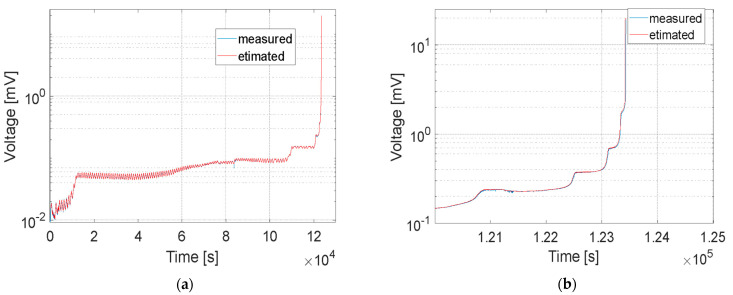
Measured and estimated data of (PU) sensor: (**a**) all registered samples, (**b**) high crack growth area.

**Figure 12:**
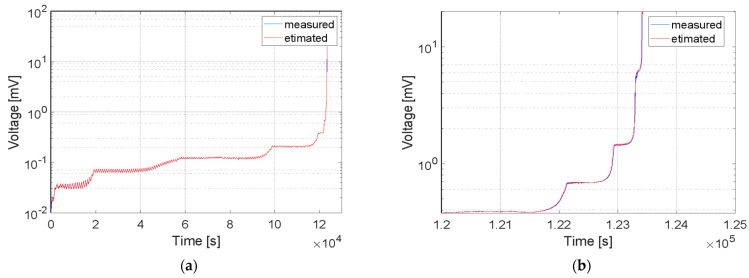
Measured and estimated data of (EP) sensor: (**a**) all registered samples, (**b**) high crack growth area.

Crack predictions with ARIMA modeling were performed. Calculation results of the designed algorithm are presented in Figure 11 and Figure 12, respectively, for the CCPS2_PU and CCPS1_EP sensors. Measured and estimated data are marked with blue and red lines, respectively.

The crack prediction was conducted with use of the ARIMA model, based on the output voltage time series registered during the research. A simple model with all parameters (*p*, *d*, *q*) equal to 1 was used. Slight discrepancies between the recorded and forecasted values of the time series were obtained. The estimated standard error was very small and had a value of less than 0.03 in the case of the MA and AR components.

The presented results show that the size of the crack could be predicted with the use of ARIMA modeling. During the analysis, 7000 iterations were made for more than 7 million samples of time series voltage. In each iteration, the ARIMA model parameters were estimated and 1000 future series values were predicted based on almost 20,000 samples. In our future work, we plan to use the estimated ARIMA model parameters as damage indicators, which would allow us to minimize the amount of archived data. In the case of the presented experiment, registered data were reduced by almost 99%. We also plan future studies of ARMIA modeling with different values of the *p*, *d*, and *q* parameters in order to verify the effectiveness of the algorithm and increase the estimation possibilities.

## 4. Discussion

The paper presents experimental verification of customized resistive crack propagation sensors, which are enhanced versions of commercially available crack gauges. Instead of using a copper or constantan measuring grid of fixed and unmodifiable shape, silver conductive paint was used on an epoxy or polyurethane insulating layer for sensing grid size adaptation. This feature enabled us to design a sensor adjusted to the specific monitored structure and install it as close to the “hot spot” as possible to detect cracks of even small dimensions.

CCPS sensors manufactured with the direct-write technique are an analogy for classic crack gauges, but combined with the Wheatstone bridge circuit. Therefore, the sensor can be utilized with a standard bridge acquisition unit instead of a strain gauge. A bridge is a more precise circuit for measuring small changes in resistance than more commonly used voltage dividers, as in [16]. Additionally, compensation for temperature and strain can be included. Data can be used for diagnostics by establishing signal thresholds for each strand breakage, based on the designed sensor resistance characteristic. With this sensor type, an equivalent crack propagation rate could be estimated in real time, due to the parallel conductive strands’ connection and the stepping sensor output characteristic. The sensors investigated in [18,19,20] are in the form of a continuous sheet, so the temperature and strain effects cannot be distinguished from the output signal without additional equipment. Furthermore, the use of carbon black or carbon nanotubes for the sheet sensor prevents visual inspection of the area under the sensor. In this paper, transparent driving layers were used for this purpose. The customized resistive crack propagation sensor proved its ability to detect and quantify fatigue cracks during the performed tests as the sensing layer broke down evenly with the structure.

Regarding insulating materials, both epoxy (EP) and polyurethane (PU) coatings showed good performance during the fatigue test, with slightly higher signal fluctuations for PU. Additional investigation is needed to establish the long-term stability of the driving layer, with a focus on strain transfer from the structure to the sensing layer as well as, e.g., bonding line degradation due to environmental factors.

The ARIMA time series analysis was revealed to be a convenient method for analyzing the data from the proposed sensors. The sufficient estimation and forecast horizon can be tuned depending on the application and crack propagation rate. Moreover, even with a relatively short forecast parameter, this method could be used to significantly decrease the amount of data that need to be archived (compared to the time domain signal). Within the investigation, more than 7 million data points for one measurement channel were processed with 7 thousand iterations of the ARIMA algorithm. If the full ARIMA output for each iteration is archived, there are 16 parameters from each iteration (but only four are needed for signal reconstruction, if a low level of estimation error is maintained).

In the approach presented in the paper, the technique is adopted to monitor fatigue cracks in metal structures (e.g., aircrafts, infrastructure, automotive, etc.), as composites are characterized by different types of fatigue failure. However, its future potential can be enhanced by targeting the work, e.g., to monitor the bond line edge of composite patch repair of the structure or to detect damages from the impacts of composite structures with an embedded sensing layer composed of a 2D conductive grid.

## Data Availability

The data presented in this study are available on request from the corresponding author.
